# Comparison of Diabetic and Non-diabetic Human Leukocytic Responses to Different Capsule Types of *Klebsiella pneumoniae* Responsible for Causing Pyogenic Liver Abscess

**DOI:** 10.3389/fcimb.2017.00401

**Published:** 2017-09-07

**Authors:** I. Russel Lee, Ethel Sng, Kok-Onn Lee, James S. Molton, Monica Chan, Shirin Kalimuddin, Ezlyn Izharuddin, David C. Lye, Sophia Archuleta, Yunn-Hwen Gan

**Affiliations:** ^1^Department of Biochemistry, Yong Loo Lin School of Medicine, National University of Singapore Singapore, Singapore; ^2^Department of Medicine, Yong Loo Lin School of Medicine, National University of Singapore Singapore, Singapore; ^3^Division of Infectious Diseases, University Medicine Cluster, National University Health System Singapore, Singapore; ^4^Communicable Disease Center, Institute of Infectious Diseases and Epidemiology, Tan Tock Seng Hospital Singapore, Singapore; ^5^Lee Kong Chian School of Medicine, Nanyang Technological University Singapore, Singapore; ^6^Department of Infectious Diseases, Singapore General Hospital Singapore, Singapore

**Keywords:** *Klebsiella pneumoniae*, liver abscess, neutrophils, neutrophil extracellular trap, peripheral blood mononuclear cells, cytokines, type 2 diabetes, hypervirulent

## Abstract

The major risk factor for Klebsiella liver abscess (KLA) is type 2 diabetes mellitus (DM), but the immunological mechanisms involved in the increased susceptibility are poorly defined. We investigated the responses of neutrophils and peripheral blood mononuclear cells (PBMCs) to hypervirulent *Klebsiella pneumoniae* (hvKP), the causative agent of KLA. DNA and myeloperoxidase levels were elevated in the plasma of KLA patients compared to uninfected individuals indicating neutrophil activation, but diabetic status had no effect on these neutrophil extracellular trap (NET) biomarkers in both subject groups. Clinical hvKP isolates universally stimulated KLA patient neutrophils to produce NETs *ex vivo*, regardless of host diabetic status. Ability of representative capsule types (K1, K2, and non-K1/K2 strains) to survive intra- and extra-cellular killing by type 2 DM and healthy neutrophils was subsequently examined. Key findings were: (1) type 2 DM and healthy neutrophils exhibited comparable total, phagocytic, and NETs killing against hvKP, (2) phagocytic and NETs killing were equally effective against hvKP, and (3) hypermucoviscous K1 and K2 strains were more resistant to total, phagocytic, and NETs killing compared to the non-mucoviscous, non-K1/K2 strain. The cytokine response and intracellular killing ability of type 2 DM as well as healthy PBMCs upon encounter with the different capsule types was also examined. Notably, the IL-12–IFNγ axis and its downstream chemokines MIG, IP-10, and RANTES were produced at slightly lower levels by type 2 DM PBMCs than healthy PBMCs in response to representative K1 and non-K1/K2 strains. Furthermore, type 2 DM PBMCs have a mild defect in its ability to control hvKP replication relative to healthy PBMCs. In summary, our work demonstrates that type 2 DM does not overtly impact neutrophil intra- and extra-cellular killing of hvKP, but may influence cytokine/chemokine production and intracellular killing by PBMCs.

## Introduction

A new, hypervirulent variant of *K. pneumoniae* (hvKP), which typically presents as community-acquired pyogenic liver abscess with sepsis, is an emerging infectious disease that poses an urgent threat to human health. First reported in Asia, Klebsiella liver abscess (KLA) is now increasingly recognized in Western countries. The vast majority of hvKP that cause KLA are K1 and K2 capsule types, accounting for 59–69 and 5–20%, respectively, of the cases described in Taiwan, Korea, Singapore, and China (Fung et al., 2002; Chung et al., 2007; Qu et al., 2015; Lee et al., 2016). In these countries, diabetes mellitus (DM) is the most commonly associated comorbidity, present in 40–78% of KLA patients (Fung et al., 2002; Chung et al., 2007; Qu et al., 2015; Lee et al., 2016).

While the most widespread and virulent capsule type K1 hvKP is capable of causing KLA in healthy individuals, non-K1 (in particular, non-K1/K2) strains tend to be restricted to patients with predisposing conditions such as DM (Fang et al., 2007; Lee et al., 2016). Risk factors for the development of KLA-associated metastatic complications such as endophthalmitis are less clear. While Fung et al. *first* reported that DM is a significant host susceptibility factor for metastatic complications (Fung et al., 2002), Fang et al. later reiterated that the bacterial virulence factor K1 alone (independent of host underlying disease) is sufficient for causing complications (Fang et al., 2007). Beyond the general understanding that DM is a risk factor for development of KLA and possibly KLA-associated complications, little is known about how DM affects the innate immune system in response to the different capsule types.

Polymorphonuclear leukocytes, most prominently neutrophils, are the major cell types in the innate immune response against bacterial pathogens. Neutrophils are crucial for early-stage defense against *K. pneumoniae* in mouse infection models (Fukutome et al., 1980; Rehm et al., 1980). Not surprisingly, humans with neutropenia are more susceptible to severe or fatal infections by *K. pneumoniae* (Valdez et al., 2009; Satlin et al., 2013). Myeloperoxidase (MPO) and neutrophil elastase (NE) have been shown to be involved in killing of *K. pneumoniae* (Belaaouaj et al., 1998; Hirche et al., 2005). Both MPO and NE localize to neutrophil extracellular traps (NETs) consisting of a meshwork of web-like DNA structures decorated with granular proteins, which are released when activated neutrophils undergo “NETosis” (Brinkmann et al., 2004; Brinkmann and Zychlinsky, 2007). NETs exhibit antimicrobial functions by trapping and killing extracellular pathogens in the blood and tissues during infection (Brinkmann et al., 2004; Brinkmann and Zychlinsky, 2007). However, *K. pneumoniae* has evolved mechanisms to evade innate host defense. For example, a recent study showed that a clinical isolate of K1 capsule type could delay neutrophil apoptosis and remained viable up to 24 h after infection, whereas infection of its acapsular mutant was cleared within 12 h and resulted in neutrophil apoptosis (Lee et al., 2017). Another recent study demonstrated that there was limited binding and uptake of carbapenem-resistant ST258 (capsule types K1/K2) isolates by neutrophils, and consequently limited killing of bacteria (Kobayashi et al., 2016).

Cytokines, which are largely produced by peripheral blood mononuclear cells (PBMCs), also play a major role in determining the outcome of bacterial infections. For instance, *K. pneumoniae* strains that elicit low TNFα production by stimulated human PBMCs were linked with early death in mice upon intraperitoneal infection compared with high-TNF-producing isolates (Pantelidou et al., 2015). Furthermore, overexpression of IL-17 in a mouse pulmonary compartment resulted in local induction of TNF, IL-1β, and G-CSF, augmented polymorphonuclear leukocytes recruitment, and enhanced bacterial clearance and survival after intratracheal challenge with *K. pneumoniae* (Ye et al., 2001). Indeed, optimal clearance of *K. pneumoniae* from host lungs requires TNF and IL-17; inflammatory monocytes were rapidly recruited to the lungs of *K. pneumoniae-*infected mice and produced TNFα, which significantly enhanced the frequency of IL-17-producing innate lymphoid cells (Xiong et al., 2016). Monocyte depletion or TNF deficiency impaired IL-17-dependent resolution of mouse pneumonia (Xiong et al., 2016).

In this study, we examined the response of neutrophils and PBMCs to KLA infection by measuring neutrophil activation biomarkers and cytokines from the plasma of KLA patients. We also isolated neutrophils from KLA and non-KLA individuals with and without type 2 DM, to assess the ability of neutrophils to kill different hvKP capsule types. In addition, we isolated PBMCs from diabetic and non-diabetic individuals to measure cytokine production as well as to examine bactericidal activity against different hvKP capsule types. We found that host diabetic status does not affect neutrophil killing of hvKP; however, it modulates PBMCs response to hvKP.

## Materials and methods

### Bacterial strains and culture conditions

KLA isolates were cultured aerobically with shaking in lysogeny broth (LB) at 37°C for ~16 h, and stationery phase bacteria were utilized for all experiments. The K1 strain SGH04, K2 strain NUH14, and K28 strain NUH29 have previously been characterized by our group (Lee et al., 2016). The other K1 strain ESKPK1 has not been characterized.

### Collection of clinical information and blood samples from patients with KLA

KLA patients recruited in this study are participants of the Antibiotics for Klebsiella Liver Abscess Syndrome Study (A-KLASS) clinical trial ongoing across three academic medical centers in Singapore (Molton et al., 2013). The primary aim of the prospective randomized A-KLASS trial is to compare the efficacy of early step-down to oral antibiotics, to continuing 4 weeks of intravenous antibiotics. Inclusion criteria include: inpatients, age ≥ 21 years, abdominal imaging suggestive of liver abscess and positive blood or abscess fluid culture for *K. pneumoniae*. Exclusion criteria include: polymicrobial liver abscess and women who are pregnant or breastfeeding. Clinical data were recorded at study entry. Blood samples (heparin-anticoagulated) were collected directly before the start of A-KLASS treatment (Day 0 visit) and 1 month after treatment (Day 28 visit).

### Collection of clinical information and blood samples from uninfected individuals

Non-diabetic individuals [normal fasting blood glucose and glycated hemoglobin (HbA1c) levels] and diabetic individuals with poor glycemic control (HbA1c > 8.5%) were also enrolled in the present study independent of the A-KLASS trial. All volunteers had no signs of infection 4 weeks before the study. Clinical data were recorded at study entry and blood samples (heparin-anticoagulated) were subsequently collected.

### Preparation of pooled human serum

Non-diabetic sera from eight healthy individuals and type 2 DM sera from eight diabetic individuals with poor glycemic control were obtained by centrifugation of whole blood at 1,000 g for 20 min at room temperature. The non-diabetic serum and the type 2 DM serum were pooled separately, and stored in aliquots at –80°C until required.

### Neutrophil killing assays

Neutrophils from human blood were isolated by density gradient centrifugation using Histopaque®-1119 and -1077 (Sigma-Aldrich), followed by hypotonic lysis to remove residual erythrocytes. Viability was > 95% as determined by trypan blue exclusion. Concentration was adjusted to 2 × 10^6^ cells/mL in RPMI 1640 + 10% pooled non-diabetic human serum or RPMI 1640 + 10% pooled diabetic human serum, and 500 μL was added into microcentrifuge tubes. Neutrophils were then treated with: (i) 20 μM cytochalasin D, (ii) 100 U/mL DNase I, (iii) 20 μM cytochalasin D + 100 U/mL DNase I, or (iv) left untreated, for 30 min at 37°C on a MACSmix™ tube rotator revolving at ~12 rpm. Bacterial strains (OD_600_ = 0.5) in RPMI + 10% pooled non-diabetic human serum or RPMI + 10% pooled diabetic human serum were added into tubes (i–iv) at a multiplicity of infection (MOI) of 1:1, and incubated at 37°C for 2 h on the tube rotator. Cells were subsequently lysed with 500 μL 0.4% Triton X-100 and viable bacteria were quantified by plating serial dilutions on LB agar. As controls, bacterial inoculum at the 0-h time point and bacterial growth in infection media without neutrophils at the 2-h time point were also quantified.

### Immunofluorescence microscopy of NETs

Neutrophils were seeded on 0.01% poly-_L_-lysine coated coverslips placed in 24-well culture plate filled with 500 μL RPMI + 10% pooled non-diabetic human serum or RPMI + 10% pooled diabetic human serum, and allowed to adhere for 30 min at 37°C with 5% CO_2_. Neutrophils were then infected with KLA isolates at an MOI of 1:1 with or without DNase I for 2 h, or left uninfected, followed by fixation with 4% paraformaldehyde. Coverslips were then washed with PBS + 10 mM glycine, and blocked with 5% BSA. Primary and secondary antibodies were diluted 1:100; coverslips were incubated with mouse anti-Klebsiella spp. (Invitrogen) and rabbit anti-NE (Abcam), followed by Alexa Fluor 633-conjugated goat anti-mouse (Thermo Fisher Scientific), Pacific Blue-conjugated goat anti-rabbit (Thermo Fisher Scientific) and 5 μM Sytox Green (Thermo Fisher Scientific). Subsequently, coverslips were washed and mounted with ProLong Gold Antifade reagent (Thermo Fisher Scientific). Images were acquired on a Zeiss LSM 510 Meta Axiovert 200M confocal microscope equipped with an LSM Zen 2009 software for image acquisition using a EC Plan-Neofluar 100 × /1.3 oil objective lens. The following excitation and emission filters were used: red fluorescence (Ex 633; Em LP 650), green fluorescence (Ex 488; Em BP 515–565), and blue fluorescence (Ex 405; Em BP 420–480). Images were formatted and analyzed using the Zen 2009 Light Edition software.

### Quantification of DNA, MPO, and cytokines in plasma samples

Plasma from human blood were isolated by density gradient centrifugation using Histopaque® -1119 and -1077. DNA was extracted from plasma using the DNA Isolation Kit—Plasma/Serum (Abcam), and plasma DNA concentration was determined using Quant-iT PicoGreen dsDNA Assay Kit (Thermo Fisher Scientific). Plasma MPO concentration was determined using Human Myeloperoxidase Quantikine ELISA Kit (R&D Systems). Plasma IL-22 and IL-23 concentrations were determined using eBioscience ELISA Ready-Set-Go Kits (Thermo Fisher Scientific). All four kits were used according to manufacturer's instructions. Plasma IL-1β, IL-6, IL-8, IL-10, IL-17A, TNF, TNFRI, TNFRII, TGF-β, MCP-1, G-CSF, GM-CSF, IFNγ, MIG, IP-10, and RANTES concentrations were determined using Cytometric Bead Array (CBA; see below).

### Bacterial infection of PBMCs

PBMCs from human blood were isolated by density gradient centrifugation using Histopaque® -1119 and -1077. Viability was >95% as determined by trypan blue exclusion. Concentration was adjusted to 1 × 10^6^ cells/mL in RPMI + 10% pooled non-diabetic human serum or RPMI + 10% pooled diabetic human serum or RPMI + 10% fetal bovine serum (FBS), and 1 mL was added into wells of a 12-well culture plate. PBMCs were allowed to acclimatize in the 37°C incubator with 5% CO_2_ for 1 h. For intracellular bacterial counts, strains (OD_600_ = 1.0) in RPMI + 10% pooled non-diabetic human serum or RPMI + 10% pooled diabetic human serum were added into wells at an MOI of 50:1, and plate was briefly centrifuged at 500 *g* for 5 min. After incubation at 37°C for 2 h, 300 μg/mL kanamycin was added to kill off extracellular bacteria. Wells were washed and PBMCs were lysed with 0.2% Triton X-100 at 2-, 6-, and 22-h post-addition of antibiotics. Viable intracellular bacteria were quantified by plating serial dilutions on LB agar. For stimulation of PBMCs to quantify cytokine production, bacterial strains (OD_600_ = 1.0) in RPMI + 10% FBS were added into wells at an MOI of 10:1, and plate was briefly centrifuged at 500 g for 5 min. After incubation at 37°C for 2 h, 300 μg/mL kanamycin was added to kill off extracellular bacteria. Plate was further incubated for 22 h before supernatant was collected into microcentrifuge tubes. Tubes were centrifuged at 5,000 g for 5 min, and 200 μL of top layer supernatant were collected for CBA.

### Cytometric bead array

Concentrations of IL-1β, IL-6, IL-10, IL-17A, TNF, TNFRI, TNFRII, TGF-β, G-CSF, GM-CSF, IL-12p70, IL-12/IL-23p40, IFNγ, MIG, IP-10, RANTES, IL-8, and MCP-1 from PBMCs-infected supernatant or plasma samples were quantitatively determined by CBA (BD Biosciences) according to manufacturer's instructions. Briefly, 18 bead populations with distinct fluorescence intensities were coated with capture antibodies specific for the above-mentioned cytokines/chemokines and receptor proteins. To identify the different bead populations, the particles containing fluorescence dyes were excited with a 638 nm laser and measured with 670/14 and 780/60 bandpass filters on an Attune NxT flow cytometer (Thermo Fisher Scientific). Capture beads were mixed with phycoerythrin (PE)-conjugated detection antibodies and incubated with recombinant standards or test samples to form sandwich complexes. Following data acquisition on the flow cytometer, results were analyzed using FCAP Array™ Software v3.0. Standard curves were set up for each individual set of reagents, and the limit of detection for each analyte is ~10 pg/mL.

### Statistical analysis

Statistical tests were performed using GraphPad Prism 6, with the use of Student's *t-*test and ANOVA as appropriate. *p-*value of < 0.05 was considered statistically significant.

### Ethics statement

All protocols and the associated informed consent documents were reviewed/approved by the National University of Singapore Institutional Review Board, National Healthcare Group Domain Specific Review Board, SingHealth Centralized Institutional Review Board and Health Science Authority, Singapore. Study procedures were conducted in accordance with the approved guidelines and regulations, and written informed consent was obtained from all participants.

## Results

### hvKP induces NET formation *Ex vivo* by neutrophils isolated from KLA patients

**Figure 1:**
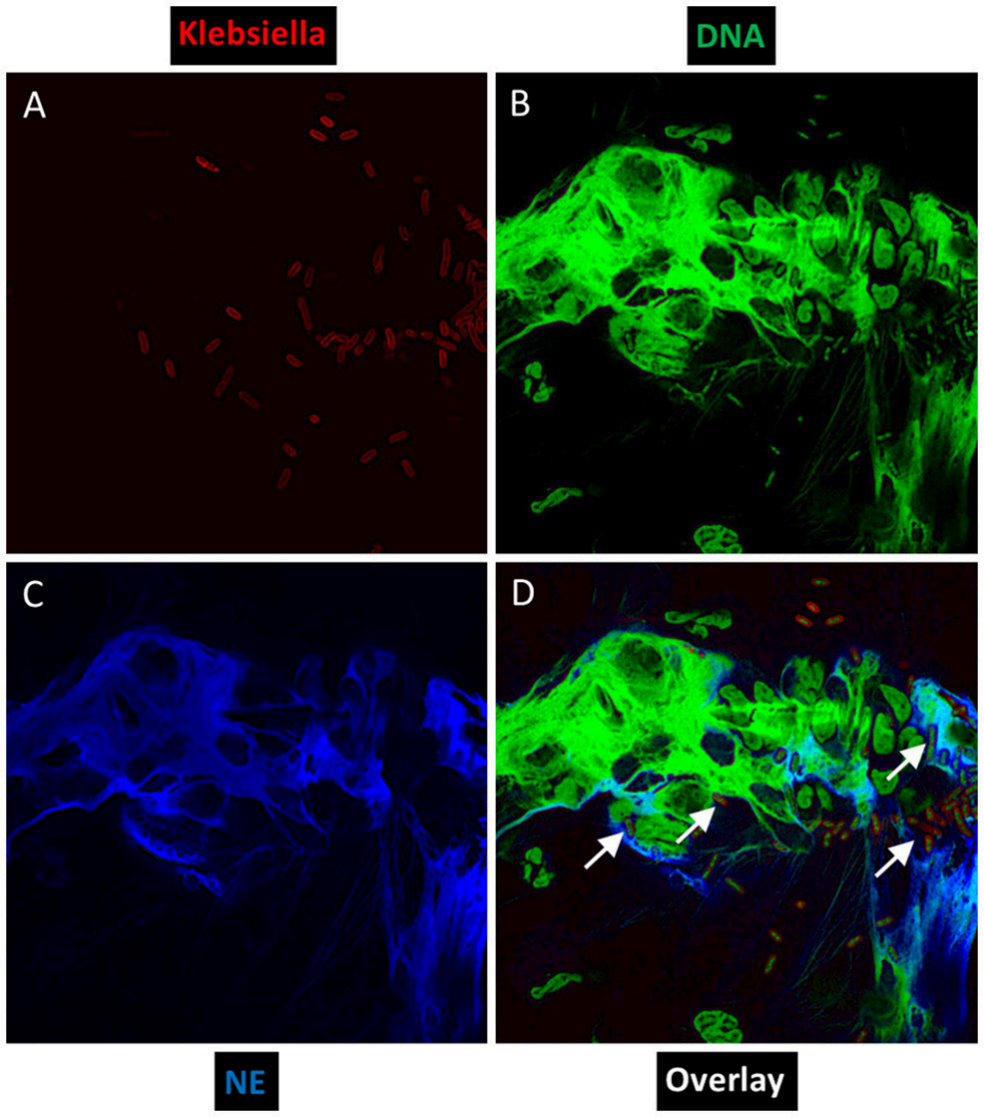
*Ex vivo* NET formation by neutrophils isolated from a KLA patient after stimulation with hvKP. Neutrophils were exposed to a capsule type K1 strain at an MOI of 1:1 for 2 h, and representative immunofluorescence images were taken at a magnification of 100×. **(A)** Bacteria were stained red with mouse anti-Klebsiella spp. (primary) and Alexa Fluor 633-conjugated goat anti-mouse (secondary). **(B)** DNA was stained green with Sytox Green. **(C)** Human neutrophil elastase (NE) was stained blue with rabbit anti-NE (primary) and Pacific Blue-conjugated goat anti-rabbit (secondary). **(D)** To visualize co-localization of NET components and hvKP, images from each channel were merged. White arrows point to physical entrapment of hvKP within NETs.

Through confocal microscopy, we investigated whether an hvKP isolate ESKPK1 can stimulate NET formation *ex vivo* independent of a priming agent such as phorbol 12-myristate 13-acetate, using neutrophils isolated from three different KLA patients. As shown in the representative images in Figure 1 from one patient, we visualized physical entrapment of hvKP within NETs produced by the human neutrophils after 2 h of infection. When an identical experiment was conducted in the presence of DNase I which degrades NETs, or when neutrophils were left uninfected, no staining could be seen confirming that the observed extracellular structures represented NETs. Therefore, like *Staphylococcus aureus*, group A Streptococcus, *Pseudomonas aeruginosa, Aspergillus fumigatus*, and *Candida albicans* (Sumby et al., 2005; Urban et al., 2006; Jaillon et al., 2007; Pilsczek et al., 2010; Young et al., 2011), hvKP can directly induce unprimed neutrophils to spontaneously form NETs.

### Activation of neutrophils in the plasma of KLA patients

**Figure 2:**
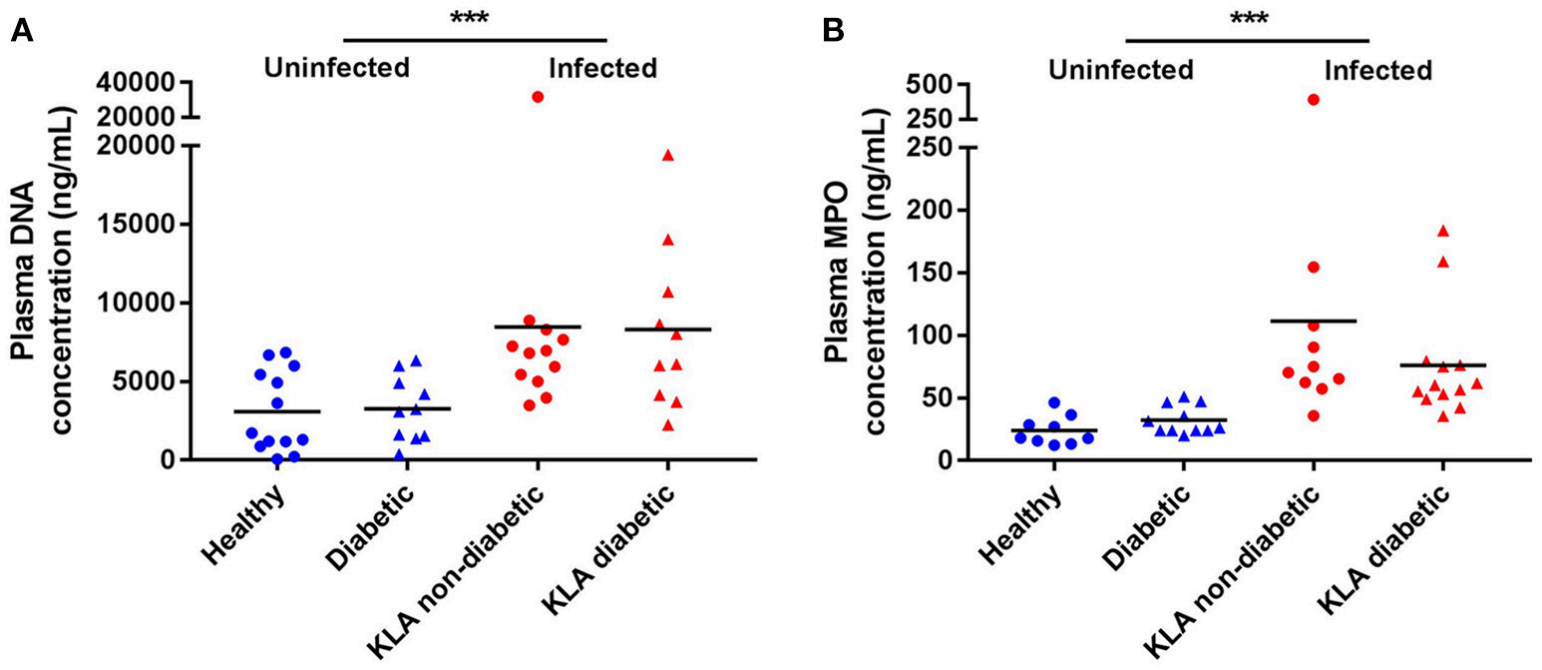
NET formation in the plasma of healthy and type 2 DM individuals as well as non-diabetic and type 2 DM KLA patients. Concentrations of DNA and MPO, which serve as biomarkers for NET formation, were determined in plasma samples. Each dot represents data from one study subject. Horizontal bars indicate the mean. SEM values are presented in text. **(A)** Healthy: mean age = 51.8 ± 11.9 years; normal fasting blood glucose and HbA1c levels. Diabetic: mean age = 56.0 ± 3.8 years; mean HbA1c = 10.1 ± 2.1%. KLA non-diabetic: mean age = 59.1 ± 16.5 years; normal fasting blood glucose and HbA1c levels. KLA diabetic: mean age = 59.2 ± 10.7 years; mean HbA1c = 10.5 ± 3.3%. **(B)** Healthy: mean age = 54.4 ± 11.3 years; normal fasting blood glucose and HbA1c levels. Diabetic: mean age = 54.6 ± 7.2 years; mean HbA1c = 9.8 ± 1.4%. KLA non-diabetic: mean age = 58.7 ± 14.3 years; normal fasting blood glucose and HbA1c levels. KLA diabetic: mean age = 59.8 ± 9.6 years; mean HbA1c = 9.8 ± 3.3%. ^***^p < 0.001.

To measure neutrophil activation in response to hvKP *in vivo*, we quantified double stranded DNA and MPO, which serve as biomarkers for NET formation, in the plasma of KLA patients when they first enrolled in the A-KLASS trial (Figure 2). Compared with age-matched uninfected individuals, DNA and MPO concentrations were significantly elevated in KLA patients (DNA: 3157.0 ± 482.3 vs. 8384.0 ± 1376.0 ng/mL, *p* = 0.0007 by Student's *t-*test; MPO: 28.4 ± 2.6 vs. 91.2 ± 16.0 ng/mL, *p* = 0.0008 by Student's *t-*test). Type 2 DM did not have an effect on neutrophil activation in uninfected individuals (DNA: 3263.0 ± 651.3 vs. 3075.0 ± 713.0 ng/mL, *p* = 0.8523 by Student's *t-*test; MPO: 32.1 ± 3.4 vs. 23.8 ± 3.9 ng/mL, *p* = 0.1223 by Student's *t-*test) or in KLA patients (DNA: 8301.0 ± 1662.0 vs. 8452.0 ± 2177.0 ng/mL, *p* = 0.9581 by Student's *t-*test; MPO: 75.9 ± 12.4 vs. 111.2 ± 33.0 ng/mL, *p* = 0.2834 by Student's *t-*test). Together, these results indicate that neutrophils play a role in defense against hvKP infection in humans, regardless of host diabetic status.

### Differential killing of hvKP capsule types by neutrophils isolated from healthy human donors

**Figure 3:**
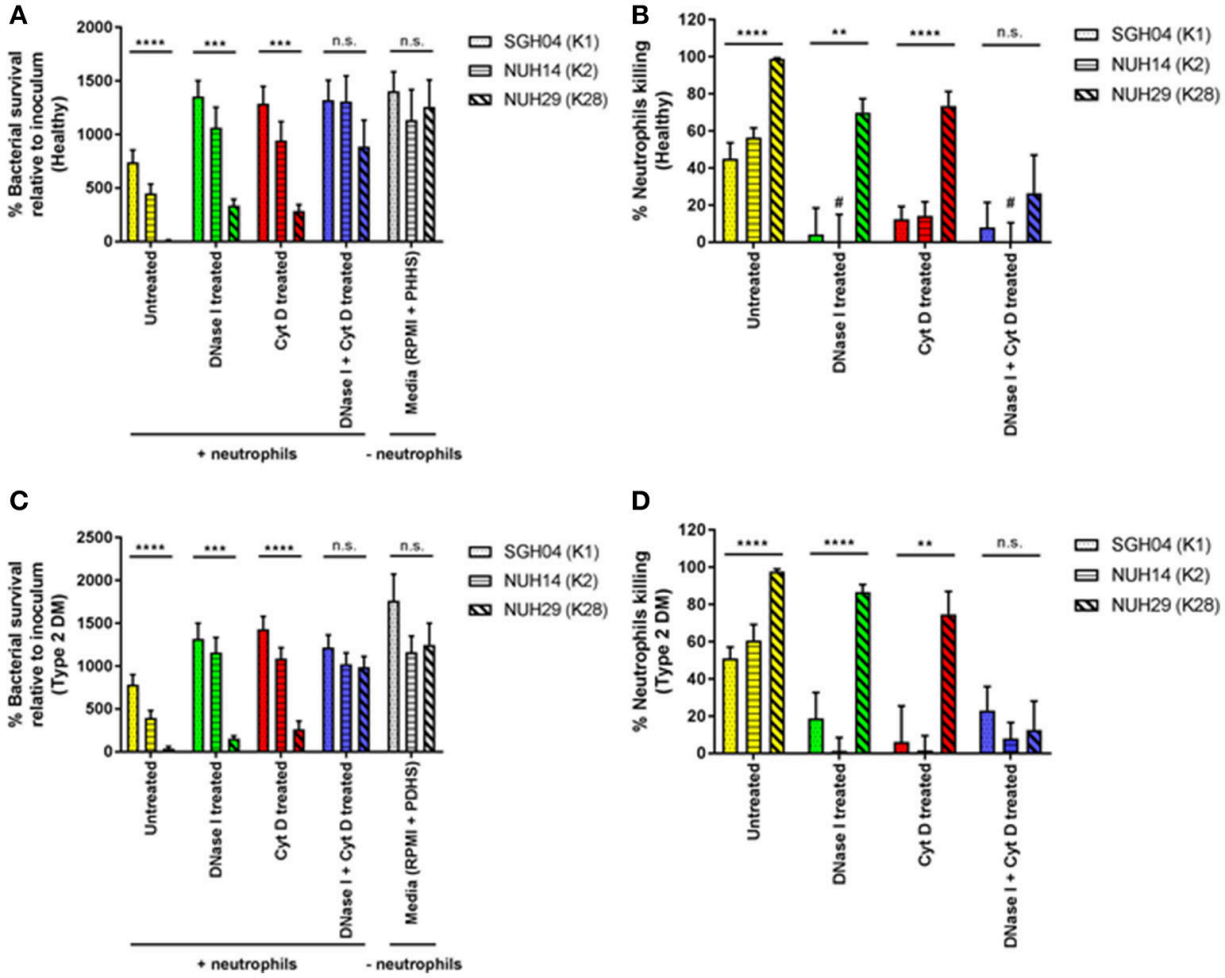
*Ex vivo* interactions between healthy as well as poorly controlled type 2 DM neutrophils and different hvKP capsule types. Neutrophils isolated from healthy donors (*n* = 8; mean age = 40.0 ± 11.7 years; normal fasting blood glucose and HbA1c levels) or type 2 DM donors with poor glycemic control (*n* = 8; mean age = 48.5 ± 13.9 years; mean HbA1c = 10.6 ± 1.7%) were pretreated with DNase I, cytochalasin D, DNase I + cytochalasin D, or left untreated. This was followed by infection with the K1 strain SGH04, K2 strain NUH14 and K28 strain NUH29. As a control, strains were also inoculated into infection media (RPMI + 10% pooled healthy human serum or RPMI + 10% pooled diabetic human serum) without neutrophils. **(A,C)** Bacterial survival was expressed as percentage change from inoculum, calculated with the equation: (CFU_+neutrophils_ at 2-h/CFU_−neutrophils_ at 0-h) × 100. **(B,D)** Percentage neutrophil killing was calculated with the equation: 100—[(CFU_+neutrophils_ at 2-h/CFU_−neutrophils_ at 2-h) × 100]. ^*^Statistical analyses in the graphs were by ANOVA. #Denotes bars with negative values that were not visually represented in the graph. Error bars represent SEM. ^**^p < 0.01, ^***^p < 0.001, ^****^p < 0.0001.

To determine whether the success of K1/K2 capsule types in causing KLA in healthy individuals is at least in part due to evasion of host innate immune response, we tested the ability of different capsule types to survive killing by healthy neutrophils. We narrowed down the panel of KLA-causing strains to three, of which two were hypermucoviscous: SGH04 (K1, ST23) and NUH14 (K2, ST2038), whereas one was non-mucoviscous: NUH29 (K28, ST20) (Lee et al., 2016). To better mimic the physiological state in the microvasculature, we maintained neutrophils and bacteria in suspension under continuous rotation, instead of employing a motionless assay on the plate. After2 h of host-pathogen interactions *ex vivo*, net survival of the K1 and K2 isolates was not significantly different from one another at 740.0 ± 115.7 and 449.7 ± 88.0%, respectively (*p* = 0.0657 by Student's *t-*test), whereas survival of the non-K1/K2 isolate was significantly lower at 11.4 ± 4.6% (*p* < 0.0001 by ANOVA) (Figure 3A). All three strains were highly serum-resistant (Lee et al., 2016), and exhibited comparable growth in infection media (RPMI supplemented with pooled healthy human serum) without neutrophils (*p* = 0.7369 by ANOVA): ~13-fold increase relative to inoculum after 2 h of incubation. Taking into consideration this 2-h proliferation in the media-only control, total neutrophil killing for the K1, K2, and non-K1/K2 isolates was 45.1 ± 8.5, 56.4 ± 5.3, and 98.9 ± 0.5%, respectively (*p* < 0.0001 by ANOVA; Figure 3B). Therefore, the K1 and K2 isolates were able to circumvent killing by healthy neutrophils to a greater extent than the non-K1/K2 isolate.

To gain insights into the mechanisms of neutrophil killing, we treated neutrophils with DNase I which dismantles NETs, cytochalasin D which blocks phagocytosis, or both DNase I and cytochalasin D, before infection with the KLA isolates. Pre-treatment with either DNase I (phagocytic killing) or cytochalasin D (NETs killing) increased the survival of the K1 and K2 isolates by ~2-fold, compared with untreated neutrophils (total killing) (Figure 3A). This suggests that phagocytosis and NETs can kill hvKP equally well under non-stationary conditions. Furthermore, it also indicates that NET-mediated killing of hvKP can occur using unprimed neutrophils, consistent with our earlier observation that hvKP can directly stimulate NET formation (Figure 1). Pre-treatment with DNase I + cytochalasin D (zero killing) did not further increase the survival of the K1 and K2 isolates, compared with treatment with DNase I or cytochalasin D alone (*p* = 0.5479 by ANOVA; Figure 3A). Therefore, inhibition of either phagocytosis or NET formation almost completely ablate the killing against the K1/K2 isolates by neutrophils (Figure 3B). K1/K2 survival after a 2-hour exposure to neutrophils pre-treated with DNase I, cytochalasin D, or DNase I + cytochalasin was comparable to growth in media control without neutrophils (*p* = 0.7140 by ANOVA; Figure 3A). Consistent with the observation of Young et al. (2011), neutrophils incapable of killing may enhance bacterial recovery relative to media control without neutrophils, which explains the “negative values” obtained in percentage neutrophil killing in some of our samples (Figure 3B).

In contrast, pre-treatment with either DNase I or cytochalasin D enhanced the survival of the non-K1/K2 isolate by ~25–30-fold compared with untreated neutrophils (Figure 3A), and killing reduced from ~99 to ~70–75% whether phagocytosis or NET formation was inhibited (Figure 3B). This reiterates that phagocytosis and NETs can kill hvKP equally well under non-stationary conditions. However, unlike the scenario with the K1/K2 isolates, pre-treatment with DNase I + cytochalasin D had a synergistic effect in reducing neutrophil killing of the non-K1/K2 isolate down to ~25% (Figure 3B). These results also indicate that the K1/K2 isolates are more resistant to phagocytic and NET-mediated killing than the non-K1/K2 isolate.

### Neutrophils isolated from poorly controlled type 2 DM individuals exhibited similar killing patterns of hvKP capsule types as healthy neutrophils

Given that type 2 DM is a risk factor for KLA development and possibly KLA-associated complications, we next tested the ability of the different capsule types to survive killing by type 2 DM neutrophils (Figures 3C,D). To maximize the chance of detecting differential killing between neutrophils from type 2 DM vs. healthy individuals, we recruited only type 2 DM individuals with poor glycemic control (HbA1c > 8.5%). As shown in Table 1, we observed comparable percentage killing of the different capsule types between neutrophils from type 2 DM and healthy individuals under all the four conditions tested (untreated, DNase I treated, cytochalasin D treated, DNase I + cytochalasin D treated). This finding was unexpected because Lin et al. had previously reported that type 2 DM neutrophils with poor glycemic control had impaired ability to phagocytose K1/K2 strains (Lin et al., 2006).

**Table 1:** Percentage killing of hvKP after exposure to neutrophils isolated from healthy donors vs. type 2 DM donors with poor glycemic control.

**Strains**	**Healthy neutrophils^a^**				**Type 2 DM neutrophils^a^**				***P-*values by Student's *t* test (Un, DI, CD, DI + CD)**
	**Un (%)**	**DI (%)**	**CD (%)**	**DI + CD (%)**	**Un (%)**	**DI (%)**	**CD (%)**	**DI + CD (%)**	
SGH04 (K1)	45.1 ± 8.5	4.3 ± 14.2	12.3 ± 7.0	7.9 ± 13.6	51.0 ± 6.1	18.8 ± 13.9	6.2 ± 19.3	23.1 ± 12.8	0.5835, 0.4812, 0.7731, 0.4348
NUH14 (K2)	56.4 ± 5.3	−0.1 ± 15.0	14.3 ± 7.5	−19.0 ± 10.6	60.8 ± 8.5	1.4 ± 7.1	1.6 ± 7.9	7.9 ± 8.7	0.6697, 0.9322, 0.2728, 0.0779
NUH29 (K28)	98.9 ± 0.5	69.9 ± 7.5	73.6 ± 7.8	26.5 ± 20.5	97.8 ± 1.2	86.8 ± 4.0	74.8 ± 12.3	12.7 ± 15.4	0.4346, 0.0734, 0.9341, 0.6007

*Un, untreated; DI, DNase I; CD, cytochalasin D*.

a *Percentage neutrophils killing was calculated with the equation: 100—[(CFU_+neutrophils_ at 2-h/CFU_−neutrophils_ at 2-h) × 100]*.

To validate this finding, we used a different hvKP K1 isolate (named ESKPK1), to separately evaluate the bactericidal activity of neutrophils from two additional groups of volunteers: (1) non-diabetic KLA patients, and (2) KLA patients with type 2 DM (good and poor glycemic control). We found that neutrophils isolated from healthy individuals, non-diabetic KLA patients and diabetic KLA patients with good or poor glycemic control all exhibited comparable total, phagocytic and NETs killing against ESKPK1 (*p* = 0.7244, 0.2324, and 0.9863, respectively, by ANOVA) (Supplementary Figure 1). Taken together, we conclude that neutrophils from individuals with type 2 DM do not display overt impairment in their killing capability against KLA isolates.

### Differential cytokine production by PBMCs from healthy and poorly controlled type 2 DM individuals in response to different hvKP capsule types

Apart from bacterial products, neutrophils in resting state can become primed by cytokines. Primed neutrophils (as well as other leukocytes) are then mobilized to the site of infection or inflammation where they encounter activating signals to trigger bacterial killing. Given that we did not observe overt impairment in type 2 DM neutrophil killing ability, we assessed whether cytokines could be differentially produced by healthy vs. type 2 DM PBMCs in response to hvKP and thus potentially influence the innate immune response to the infection. We stimulated healthy as well as poorly controlled type 2 DM PBMCs with different hvKP capsule types and measured production of 17 pro- and anti-inflammatory cytokines/chemokines and receptor proteins. Given that the hypermucoviscous K1 strain SGH04 and K2 strain NUH14 exhibited comparable responses in the neutrophil killing assays, we selected SGH04 as the representative hypermucoviscous strain and NUH29 as the representative non-mucoviscous, non-K1/K2 strain for this experiment. Infection of both healthy and type 2 DM PBMCs with SGH04 or NUH29 failed to produce detectable levels of IL-17A and TNFRI after 24-h post-infection (not shown). On the other hand, the levels of IL-1β, TNF, and GM-CSF produced by both healthy and type 2 DM PBMCs were comparable regardless of strain type (Supplementary Figure 2). For the remaining cytokines, two patterns of differential production were observed.

**Figure 4:**
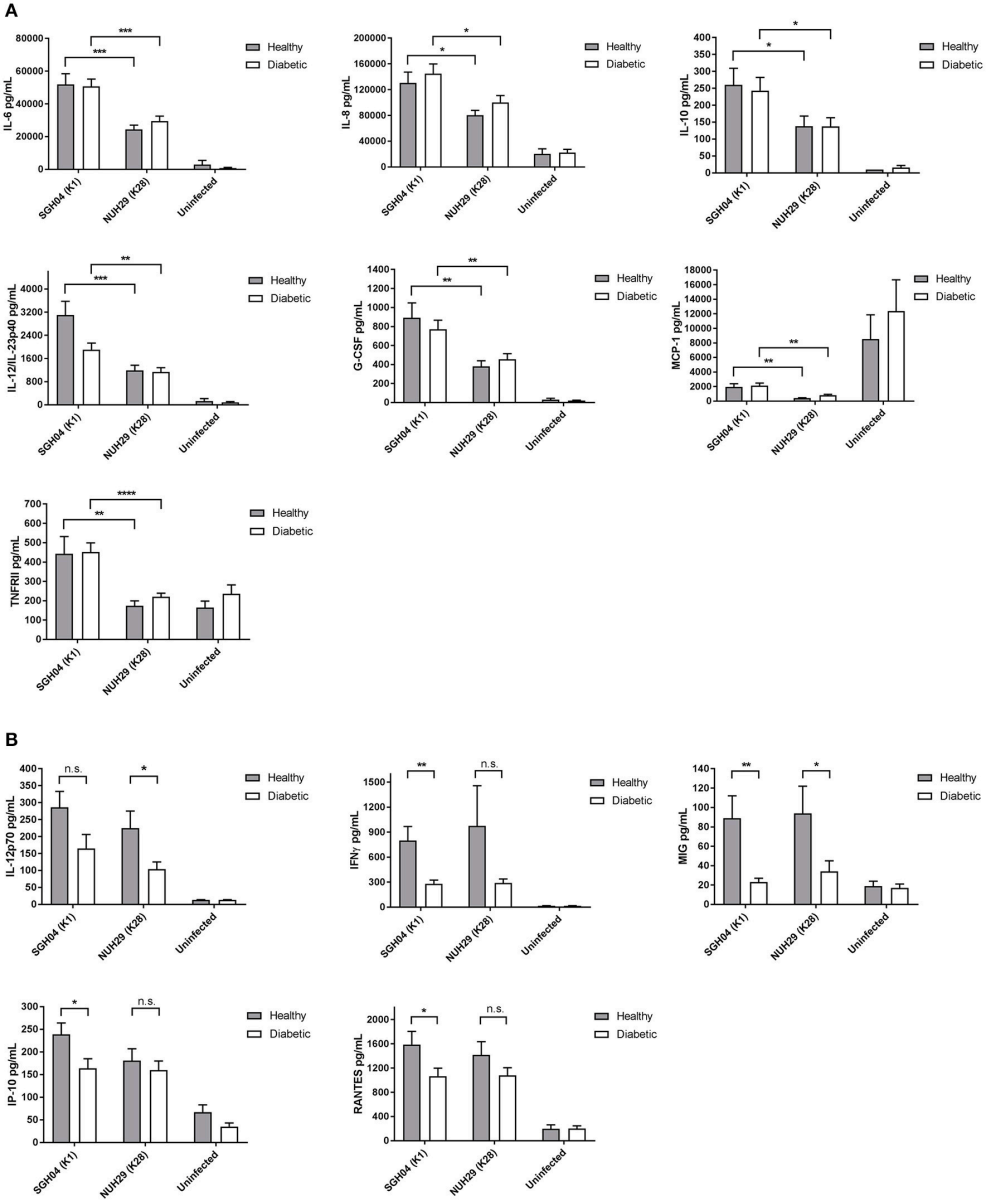
Cytokine production by healthy and poorly controlled type 2 DM PBMCs induced by various hvKP capsule types. PBMCs isolated from healthy donors (*n* = 12; mean age = 45.0 ± 12.5 years; normal fasting blood glucose and HbA1c levels) or type 2 DM donors with poor glycemic control (*n* = 16; mean age = 54.8 ± 13.7 years; mean HbA1c = 10.3 ± 1.6%) were infected with the K1 strain SGH04 and the K28 strain NUH29, or left uninfected. Kanamycin was added at 2-h post-infection to kill off extracellular bacteria and culture supernatant was collected at 24-h post-infection for CBA analysis. **(A)** The K1 isolate induced higher production of IL-6, IL-8, IL-10, IL-12/IL-23p40, G-CSF, MCP-1, and TNFRII than the non-K1/K2 isolate. **(B)** Type 2 DM PBMCs generated a slightly lower production of IL-12p70, IFNγ, MIG, IP-10, and RANTES than healthy PBMCs. Error bars represent SEM. ^*^p < 0.05, ^**^p < 0.01, ^***^p < 0.001, ^****^p < 0.0001.

In the first pattern, cytokine production was differentially induced by strain type but not affected by diabetic status of PBMCs. Specifically, the K1 isolate stimulated higher production of IL-6 (Healthy: *p* = 0.0005 by Student's *t-*test; Diabetic: *p* = 0.0004 by Student's *t-*test), IL-8 (Healthy: *p* = 0.0115 by Student's *t-*test; Diabetic: *p* = 0.0217 by Student's *t-*test), IL-10 (Healthy: *p* = 0.0498 by Student's *t-*test; Diabetic: *p* = 0.0366 by Student's *t-*test), IL-12/IL-23p40 (Healthy: *p* = 0.0008 by Student's *t-*test; Diabetic: *p* = 0.0069 by Student's *t-*test), G-CSF (Healthy: *p* = 0.0048 by Student's *t-*test; Diabetic: *p* = 0.0086 by Student's *t-*test), MCP-1 (Healthy: *p* = 0.0013 by Student's *t-*test; Diabetic: *p* = 0.0014 by Student's *t-*test), and TNFRII (Healthy: *p* = 0.0076 by Student's *t-*test; Diabetic *p* < 0.0001 by Student's *t-*test) than the non-K1/K2 isolate (Figure 4A). The observation that the K1 isolate stimulated higher pro- and anti-inflammatory cytokine production compared with the non-K1/K2 isolate suggests a different innate immune response signature against hypermucoviscous vs. non-mucoviscous hvKP strains.

In the second pattern, we observed a trend whereby cytokine production was not influenced by strain type but was generated at slightly lower levels in type 2 DM PBMCs than healthy PBMCs (Figure 4B): IL-12p70 (K1: *p* = 0.0794 by Student's *t-*test; K28: *p* = 0.0185 by Student's *t-*test), IFNγ (K1: *p* = 0.0015 by Student's *t-*test; K28: *p* = 0.0983 by Student's *t-*test), MIG (K1: *p* = 0.0039 by Student's *t-*test; K28: *p* = 0.0432 by Student's *t-*test), IP-10 (K1: *p* = 0.0303 by Student's *t-*test; K28: *p* = 0.5129 by Student's *t-*test) and RANTES (K1: *p* = 0.0394 by Student's *t-*test; K28: *p* = 0.1682 by Student's *t-*test). It is noteworthy that not all pairwise comparisons between type 2 DM vs. healthy PBMCs production of these cytokines upon stimulation with either the K1 or K28 strain achieved statistical significance. Specifically, IL-12p70 production upon stimulation with K1, and IFNγ, IP-10, and RANTES production upon stimulation with K28, did not reach statistical significance.

### PBMCs from poorly controlled type 2 DM individuals exhibited intracellular killing defect against hvKP

Since IFNγ primes phagocytes such as monocytes for increased oxidative burst while MIG/IP-10/RANTES act as chemoattractants for monocytes/macrophages, natural killer cells or T cells, we investigated whether type 2 DM PBMCs with poor glycemic control have defective capacity to control hvKP infection relative to healthy PBMCs. Unlike neutrophils which have the ability to kill pathogens via intra- and extra-cellular mechanisms, the primary mode of killing by PBMCs is through intracellular means. PBMCs from the respective groups were infected with the K1 strain SGH04 and the non-K1/K2 strain NUH29, after which kanamycin was added to kill off extracellular bacteria at 2-h post-infection, and number of intracellular bacteria were quantified at 4-, 8-, and 24-h post-infection (Supplementary Figure 3).

**Figure 5:**
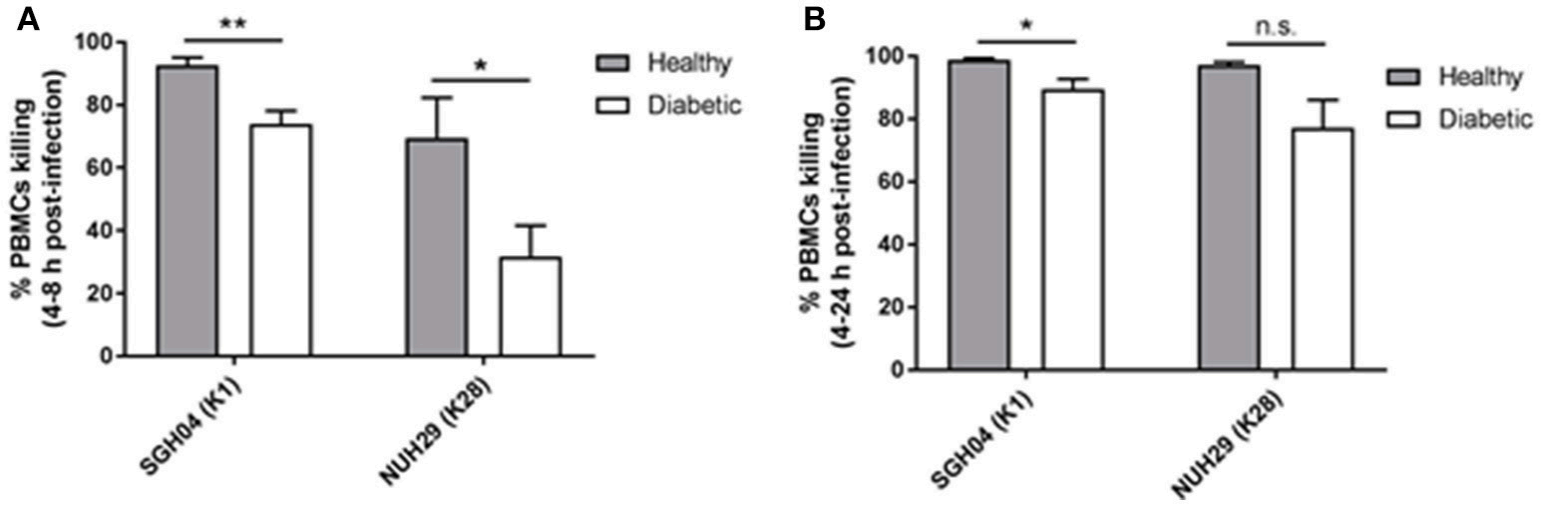
Intracellular survival of different hvKP capsule types in healthy and poorly controlled type 2 DM PBMCs. PBMCs isolated from healthy donors (*n* = 8; mean age = 58.1 ± 8.6 years; normal fasting blood glucose and HbA1c levels) or type 2 DM donors with poor glycemic control (*n* = 10; mean age = 60.3 ± 9.5 years; mean HbA1c = 9.8 ± 3.2%) were infected with the K1 strain SGH04 and the K28 strain NUH29. Kanamycin was added at 2-h post-infection to kill off extracellular bacteria, and the number of intracellular bacteria was quantified at 4-, 8-, and 24-h post-infection. **(A)** Percentage killing between 4 and 8 h post-infection. **(B)** Percentage killing between 4 and 24 h post-infection. Error bars represent SEM. ^*^p < 0.05, ^**^p < 0.01.

Between 4 and 8 h post-infection, killing by healthy PBMCs against the K1 and non-K1/K2 isolates was 92.6 ± 2.5 and 69.5 ± 12.8%, respectively, whereas killing by type 2 DM PBMCs was lower at 74.0 ± 4.1 and 31.8 ± 9.8%, respectively (*p* = 0.0031 and 0.0294, respectively, by Student's *t-*test; Figure 5A). Between 4 and 24 h post-infection, killing by healthy PBMCs against the K1 and non-K1/K2 isolates was 98.9 ± 0.5 and 97.1 ± 1.0%, respectively, whereas killing by type 2 DM PBMCs was slightly lower at 89.5 ± 3.2 and 77.1 ± 8.9%, respectively (*p* = 0.0202 and 0.0640, respectively, by Student's *t-*test; Figure 5B). Therefore, type 2 DM PBMCs generally displayed a mild defect in its ability to control hvKP intracellular replication relative to healthy PBMCs.

### Plasma TNFRI and TNFRII levels were elevated in patients with KLA

To identify biomarkers of KLA, we measured the levels of 18 cytokines/chemokines/receptor proteins in the plasma of KLA patients at the time of enrolment into the clinical trial (Day 0/1) and 1 month after the A-KLASS treatment (Day 28) as well as of healthy individuals. The plasma levels of IL-1β, IL-6, IL-8, IL-10, IL-17A, IL-22, IL-23, IFNγ, TNF, TGF-β, MCP-1, G-CSF, and GM-CSF were low even in the A-KLASS Day 0/1 samples—majority of which were below the detection limit (not shown). This suggests that immune response to the infection was generally more localized than systemic. Alternatively, peak cytokine production may have subsided; by the time patients were diagnosed with KLA and entered into the trial, they may have been on antibiotics for a maximum of 7 days.

**Figure 6:**
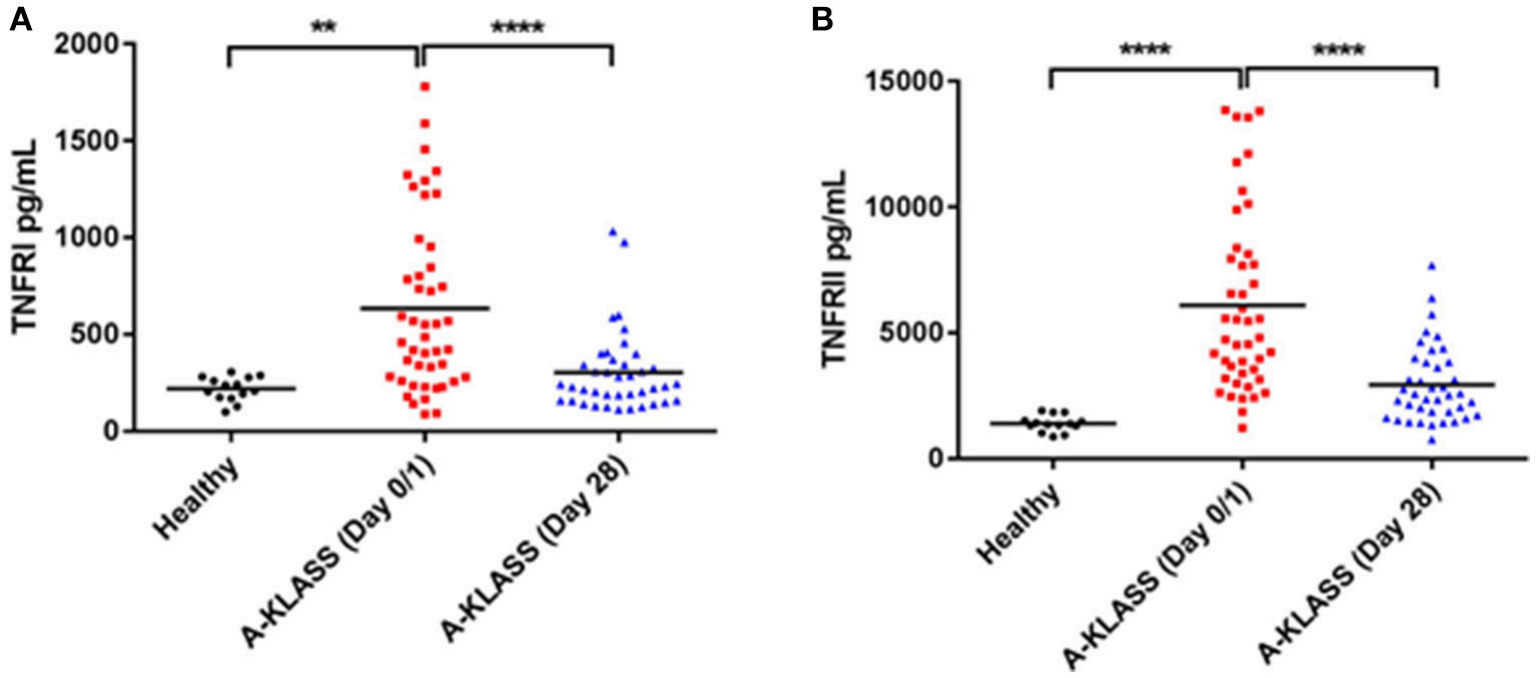
TNF receptor protein levels in the plasma of healthy individuals and KLA patients. Each dot represents data from one study subject. Horizontal bars indicate the mean. Healthy: mean age = 52.4 ± 10.7 years. A-KLASS (Day 0/1): mean age = 60.1 ± 13.0 years. A-KLASS (Day 28): mean age = 60.5 ± 13.6 years. **(A)** TNFRI levels in the plasma of healthy individuals as well as KLA patients at the time of enrollment into the clinical trial and 1 month after the A-KLASS treatment. **(B)** TNFRII levels in the plasma of healthy individuals as well as KLA patients at the time of enrollment into the clinical trial and 1 month after the A-KLASS treatment. ^**^p < 0.01, ^****^p < 0.0001.

On the other hand, the levels of MIG, IP-10, and RANTES in the plasma of healthy individuals and KLA patients were detectable but comparable (Supplementary Figure 4). However, the levels of soluble TNFRI and TNFRII were significantly higher in the plasma of KLA patients at the time of enrolment into the clinical trial (Day 0/1) compared to 1 month after the A-KLASS treatment (Day 28) or compared to healthy individuals (*p* < 0.0001 by ANOVA; Figures 6A,B). This suggests that the higher levels of TNFRI and TNFRII at the beginning of the trial decreased to levels similar to uninfected healthy controls after 28 days of antibiotic treatment.

## Discussion

Neutrophils have been documented to kill *K. pneumoniae* through NET formation in sepsis originating from pulmonary infection (Achouiti et al., 2012). In KLA mouse infection models, neutrophil infiltration into liver parenchyma had been reported (Fung et al., 2011), and postulated to be a “Trojan horse” in picking up bacteria from a liver abscess and transporting them to distant metastases (Lin et al., 2010). A recent study showed that K1 isolates could delay human neutrophil apoptosis and survive neutrophil killing, again postulating that a “Trojan horse” mechanism could also be at work in humans (Lee et al., 2017). This is in line with another recent study which demonstrated that K1/K2 capsule types of hvKP are more resistant to human neutrophil killing than “classic” *K. pneumoniae* (Wang et al., 2017), which could explain the virulence of the K1/K2 isolates in causing disease. In contrast, our understanding about the response mounted by neutrophils from diabetic subjects upon encounter with different hvKP capsule types is incomplete. Lin et al. had reported that phagocytosis of K1/K2 isolates, but not non-K1/K2 isolates, was significantly impaired in type 2 DM neutrophils with poor glycemic control relative to healthy neutrophils (Lin et al., 2006). However, it is not known whether type 2 DM neutrophils have an overall defective killing ability against different capsule types.

In this study, we demonstrated that KLA patients with active disease showed significant levels of neutrophil activation by measuring double-stranded DNA and MPO in the plasma. The measurement of these two parameters as a specific indication of NET formation has been established by several studies (Caudrillier et al., 2012; Yoo et al., 2014). We did not observe any differences in the levels of these biomarkers in relation to host diabetic status. This is consistent with our *ex vivo* data, where we isolated neutrophils from healthy and type 2 DM individuals without infection, and infected the cells with different hvKP capsule types. The neutrophils from both healthy and type 2 DM individuals showed equivalent killing of hvKP of various capsule types. Despite a report by Lin et al. identifying impaired phagocytosis of K1/K2 isolates by neutrophils from individuals with type 2 DM (Lin et al., 2006), we found that neutrophils from individuals with type 2 DM did not display defective killing against any of the capsule types. Perhaps, the impaired phagocytosis of K1/K2 isolates by neutrophils from individuals with poorly controlled type 2 DM may be compensated by efficient NET-mediated killing. Alternatively, differences in experimental conditions may explain different experimental outcomes. For example, Young et al. had reported that when neutrophils and *P. aeruginosa* were layered together on a motionless surface, phagocytic killing was the predominant response, whereas under conditions in which neutrophils and *P. aeruginosa* were maintained in suspension, nearly all killing were NET-mediated (Young et al., 2011).

We also did not observe preferential phagocytic- and NET-mediated killing of hvKP of the various capsule types by human neutrophils. Both intracellular killing (phagocytosis) and extracellular killing (NETs) appear to be equally effective against hvKP. Hence, the mechanism of neutrophil killing is likely to be pathogen-specific, given that Menegazzi et al. had reported that phagocytosis is the primary mode by which neutrophils kill *S. aureus* and *C. albicans* (Menegazzi et al., 2012). Nonetheless, it is worth noting that we performed the killing assay in suspended state of constant motion, unlike Menegazzi et al. whose assay was maintained stationary on a surface. However, consistent with other studies, killing of K1/K2 isolates was reduced compared with the non-K1/K2 isolate. Furthermore, phagocytosis and NETs had a synergistic effect on the killing of the non-K1/K2 isolate, whereas they showed no synergy in the killing of K1/K2 isolates.

While we did not observe overt impairment in type 2 DM neutrophil killing of hvKP, we noted that PBMCs from type 2 DM individuals with poor glycemic control have a subtle defect in IL-12–IFNγ production when infected with hvKP, which likely resulted in the slight reduction of downstream chemokine production including MIG, IP-10, and RANTES. IL-12 is a major inducer of IFNγ (Wysocka et al., 1995; Tan et al., 2012), while IFNγ induces MIG, IP-10, and RANTES production (Lee et al., 2000; Stassi et al., 2008; Karonitsch et al., 2009). This particular defect was highly specific because other pro-inflammatory cytokines including TNF, IL-1β, and IL-8, and anti-inflammatory cytokines such as IL-10, were produced at comparable levels by PBMCs from poorly controlled type 2 DM individuals and healthy individuals in response to hvKP. Our group had previously shown that defective IL-12–IFNγ production in *Burkholderia pseudomallei-* and *Mycobacterium tuberculosis-*infected PBMCs correlated with deficiency in intracellular glutathione (GSH) concentrations in poorly controlled type 2 DM individuals (Tan et al., 2012). This poorer IL-12–IFNγ production in type 2 DM individuals is not a general defect, as PBMCs from individuals with type 2 DM infected with *Salmonella enterica* produced normal levels of IL-12 and IFNγ (Tan et al., 2012).

A common observation of defective IL-12–IFNγ production in *B. pseudomallei-, M. tuberculosis-* and hvKP*-*infected type 2 DM PBMCs is the increased susceptibility of diabetic individuals in developing melioidosis, tuberculosis, KLA, and possibly KLA-associated complications (Fung et al., 2002; Cheng and Currie, 2005; Leung et al., 2008; Gan, 2013). The IL-12–IFNγ axis is pro-inflammatory and is critical for activating phagocytes such as monocytes/macrophages, so that they become microbicidal. Although they are known to be important for intracellular pathogen control, they have also been shown to be important for some extracellular pathogens (Cano et al., 2015; Lemon and Weiser, 2015). Our results show that PBMCs from individuals with poorly controlled type 2 DM have slightly reduced killing efficiency against hvKP compared with PBMCs from healthy individuals. IFNγ and its induction of IP-10 had been shown to be important for generating Th1 immunity that is responsible for clearance of pulmonary *K. pneumoniae* (Moore et al., 2002; Zeng et al., 2005). Therefore, PBMCs may be critical for defense against hvKP particularly during the dissemination process to vital organs such as the lung, eye, or brain, which may lead to metastatic complications and poorer clinical outcomes. Although this study did not examine the adaptive responses of type 2 DM patients to hvKP, it is possible the adaptive Th1 responses are also compromised.

We also found that IL-8 was more highly produced by PBMCs infected with the K1 isolate than the non-K1/K2 isolate. This is in agreement with the study showing that increased production of IL-8 in neutrophils delayed apoptosis and maintained viable bacteria over a longer period of time (Lee et al., 2017). Thus, higher levels of IL-8 may not only be produced by neutrophils but also PBMCs, which could have an impact on not just neutrophil chemotaxis in response to this chemokine, but in extending the life span of neutrophils as possible vehicles of metastatic spread. Generally, higher levels of pro-inflammatory cytokines were induced by the K1 isolate, which could trigger more inflammation in the patients. Higher levels of inflammation in KLA patients could also be detected when we measured plasma concentrations of cytokines/chemokines and receptor proteins, despite suboptimal timing of collection. Most cytokines were present at very low concentrations at the point the patients were enrolled in the A-KLASS trial. Prior to enrolment, most of these patients were already exposed to antibiotics. This could explain the lower levels of inflammation observed systemically. Despite this, we found that soluble TNFRI and TNFRII levels were elevated compared with healthy controls, and decreased over the course of A-KLASS treatment, showing that they are stable biomarkers of infection. This is consistent with literature reporting the correlation between inflammation and TNFRI and TNFRII in conditions such as inflammatory bowel disease and Crohn's disease (Sedger and McDermott, 2014).

In summary, we have shown that plasma soluble TNF receptors, MPO and double-stranded DNA are stable markers of KLA infection in humans. Neutrophils are activated to form NETs during KLA infection and they can kill hvKP via NETs or phagocytosis. Although type 2 DM does not cause overt impairment in neutrophil killing, it reduces Th1 cytokine production slightly by PBMCs, which could impact efficient control and clearance of the infection.

## Author contributions

IL, ES, JM, SA, and YG designed the project and YG oversaw the project. KL, JM, MC, SK, EI, DL, and SA recruited the participants. IL and ES performed the experiments. IL and YG wrote the manuscript. JM, DL, and SA reviewed the manuscript and provided recommendations.

### Conflict of interest statement

The authors declare that the research was conducted in the absence of any commercial or financial relationships that could be construed as a potential conflict of interest.

## Abbreviations

A-KLASS: Antibiotics for Klebsiella Liver Abscess Syndrome Study
CBA: Cytometric Bead Array
DM: diabetes mellitus
HbA1c: glycated hemoglobin
hvKP: hypervirulent variant of Klebsiella pneumoniae
KLA: Klebsiella liver abscess
MPO: myeloperoxidase
NE: neutrophil elastase
NETs: neutrophil extracellular traps
PBMCs: peripheral blood mononuclear cells.
