# Supplementary material for: Comparison of Diabetic and Non-diabetic Human Leukocytic Responses to Different Capsule Types of Klebsiella pneumoniae Responsible for Causing Pyogenic Liver Abscess
Source: Front Cell Infect Microbiol. 2017 Sep 7;7:401. doi: 10.3389/fcimb.2017.00401 (PMC5594087; doi:10.3389/fcimb.2017.00401)
Supplement: Supplementary file 1 [file Presentation1.PPTX]

## Slide 1
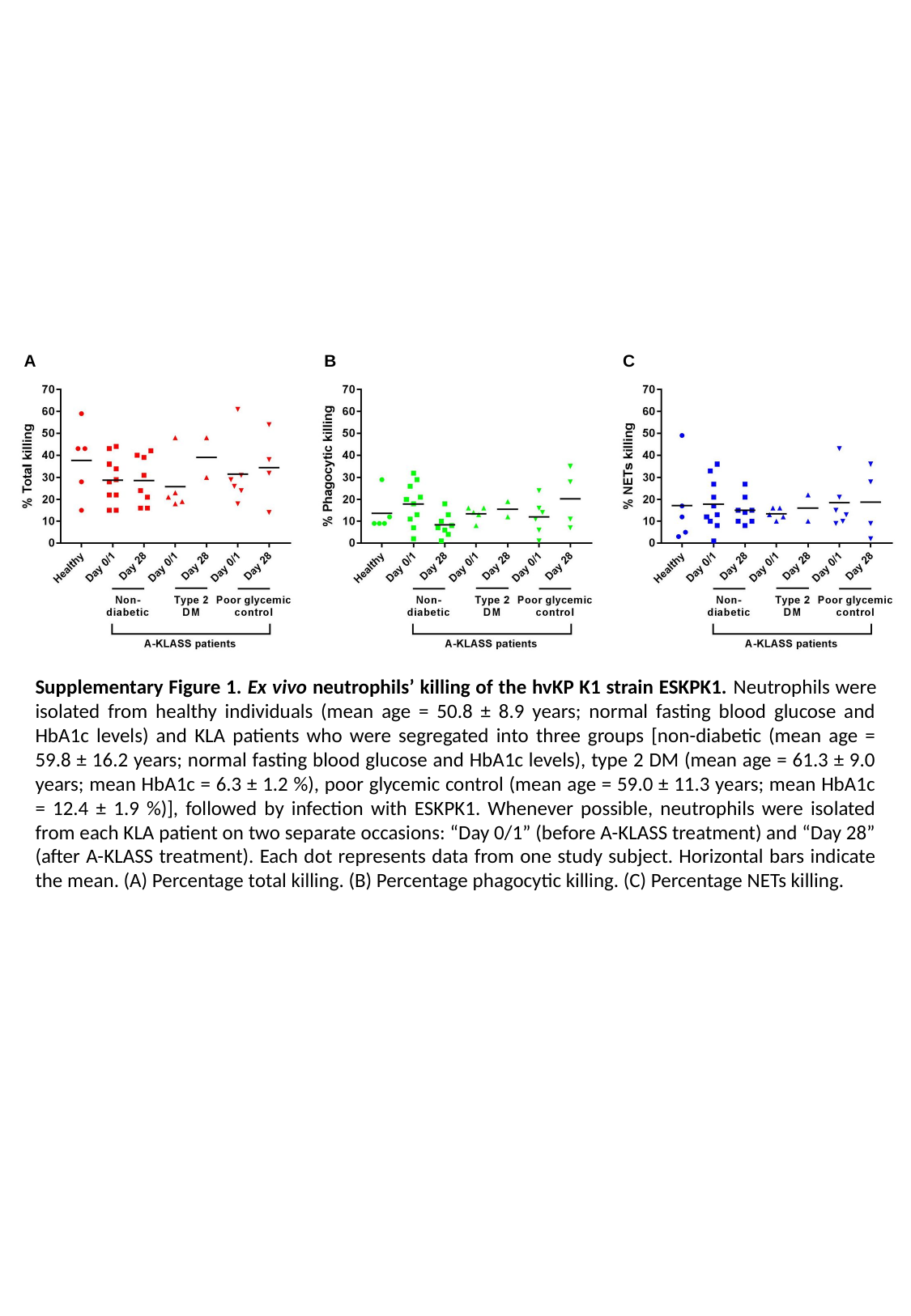

A
B
C
Supplementary Figure 1. Ex vivo neutrophils’ killing of the hvKP K1 strain ESKPK1. Neutrophils were isolated from healthy individuals (mean age = 50.8 ± 8.9 years; normal fasting blood glucose and HbA1c levels) and KLA patients who were segregated into three groups [non-diabetic (mean age = 59.8 ± 16.2 years; normal fasting blood glucose and HbA1c levels), type 2 DM (mean age = 61.3 ± 9.0 years; mean HbA1c = 6.3 ± 1.2 %), poor glycemic control (mean age = 59.0 ± 11.3 years; mean HbA1c = 12.4 ± 1.9 %)], followed by infection with ESKPK1. Whenever possible, neutrophils were isolated from each KLA patient on two separate occasions: “Day 0/1” (before A-KLASS treatment) and “Day 28” (after A-KLASS treatment). Each dot represents data from one study subject. Horizontal bars indicate the mean. (A) Percentage total killing. (B) Percentage phagocytic killing. (C) Percentage NETs killing.

## Slide 2
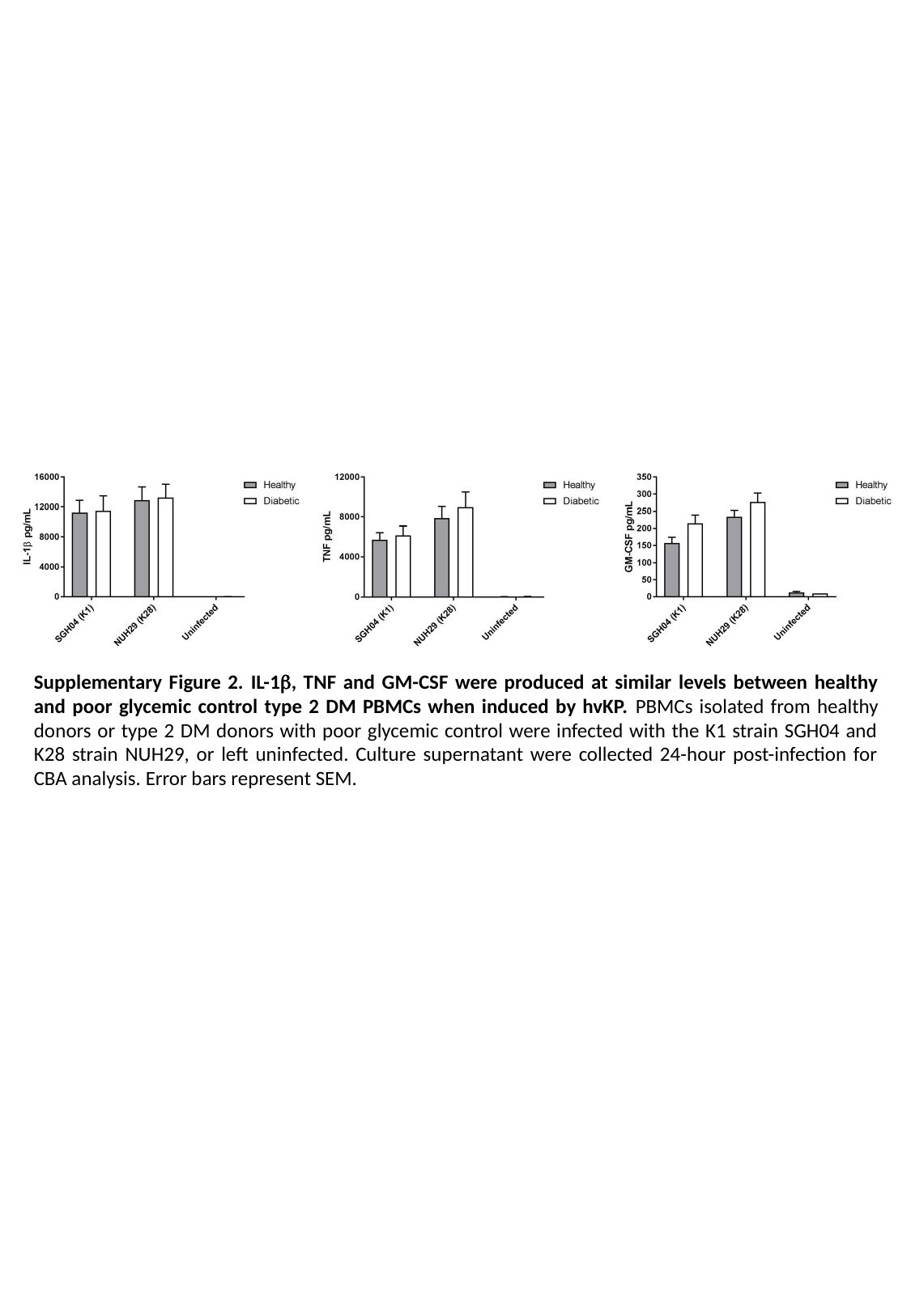

Supplementary Figure 2. IL-1β, TNF and GM-CSF were produced at similar levels between healthy and poor glycemic control type 2 DM PBMCs when induced by hvKP. PBMCs isolated from healthy donors or type 2 DM donors with poor glycemic control were infected with the K1 strain SGH04 and K28 strain NUH29, or left uninfected. Culture supernatant were collected 24-hour post-infection for CBA analysis. Error bars represent SEM.

## Slide 3
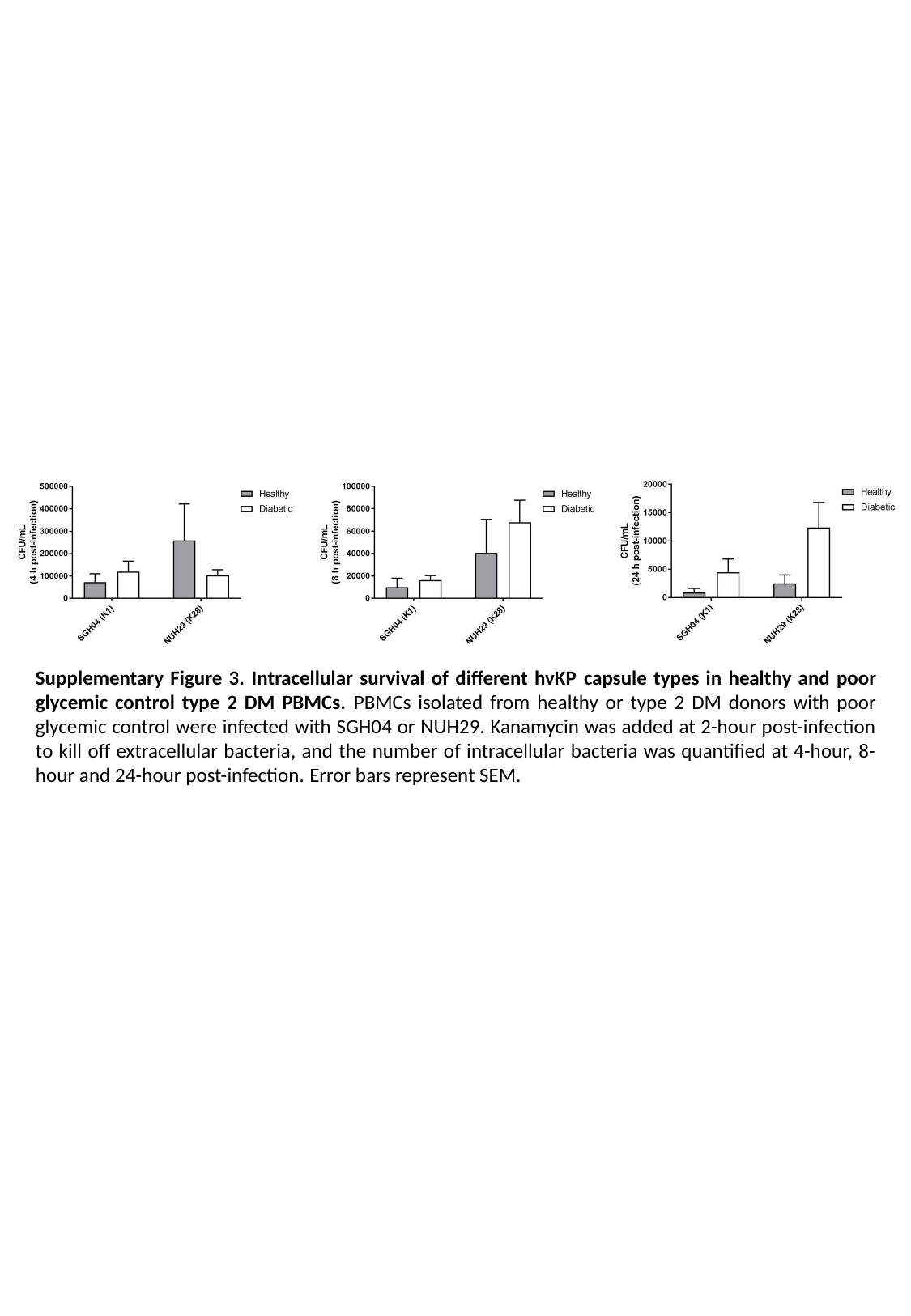

Supplementary Figure 3. Intracellular survival of different hvKP capsule types in healthy and poor glycemic control type 2 DM PBMCs. PBMCs isolated from healthy or type 2 DM donors with poor glycemic control were infected with SGH04 or NUH29. Kanamycin was added at 2-hour post-infection to kill off extracellular bacteria, and the number of intracellular bacteria was quantified at 4-hour, 8-hour and 24-hour post-infection. Error bars represent SEM.

## Slide 4
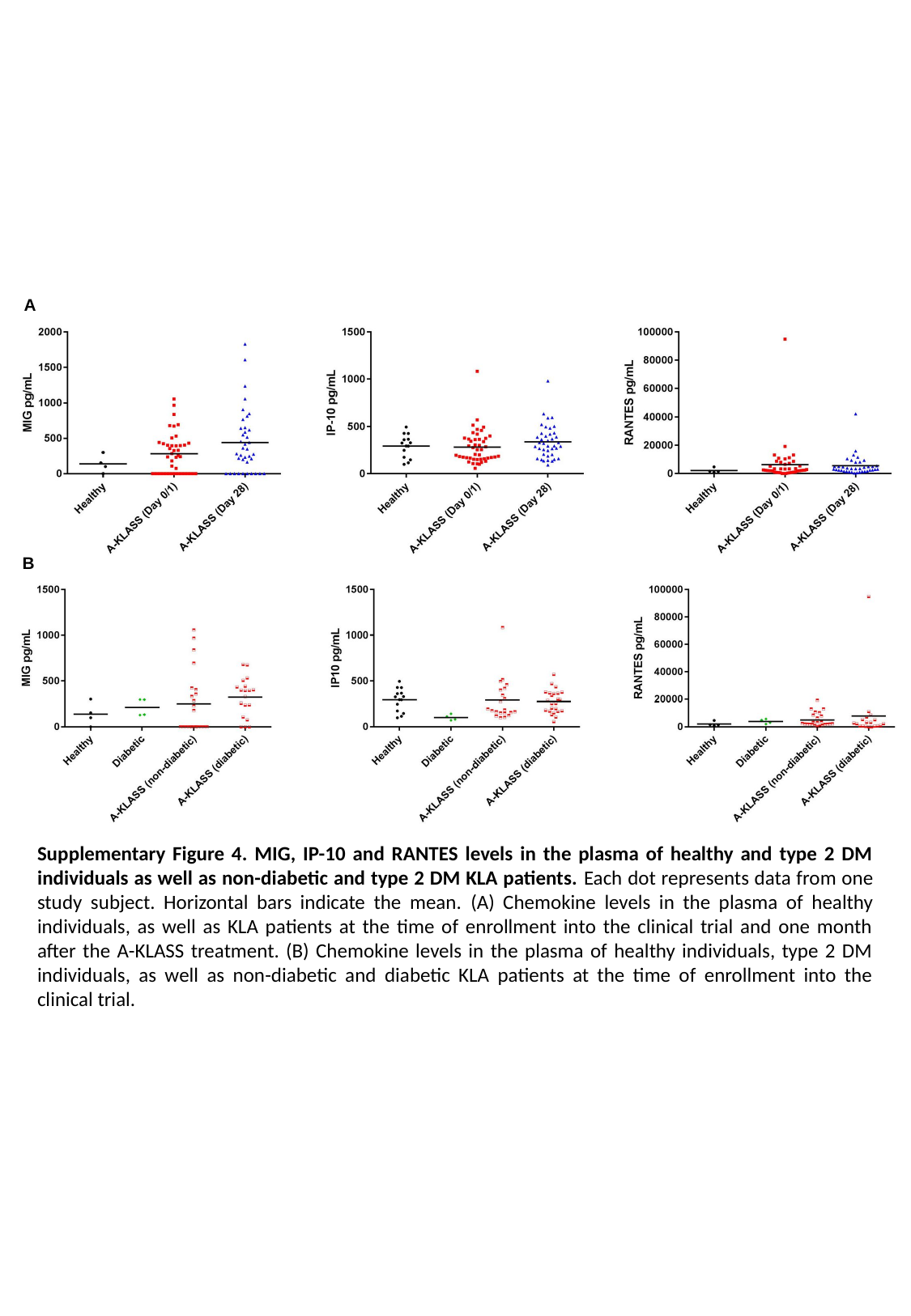

A
B
Supplementary Figure 4. MIG, IP-10 and RANTES levels in the plasma of healthy and type 2 DM individuals as well as non-diabetic and type 2 DM KLA patients. Each dot represents data from one study subject. Horizontal bars indicate the mean. (A) Chemokine levels in the plasma of healthy individuals, as well as KLA patients at the time of enrollment into the clinical trial and one month after the A-KLASS treatment. (B) Chemokine levels in the plasma of healthy individuals, type 2 DM individuals, as well as non-diabetic and diabetic KLA patients at the time of enrollment into the clinical trial.
